# Stem Cell Based Models in Congenital Hyperinsulinism – Perspective on Practicalities and Possibilities

**DOI:** 10.3389/fendo.2022.837450

**Published:** 2022-02-18

**Authors:** Väinö Lithovius, Timo Otonkoski

**Affiliations:** ^1^ Stem Cells and Metabolism Research Program, Faculty of Medicine, University of Helsinki, Helsinki, Finland; ^2^ Children’s Hospital, Helsinki University Hospital, Helsinki, Finland

**Keywords:** congenital hyperinsulinism, stem cell derived islets, disease modeling, drug screening and discovery, insulin secretion, hypoglycemia, genetic defects

## Abstract

Congenital hyperinsulinism (CHI) is a severe inherited neonatal disorder characterized by inappropriate insulin secretion caused by genetic defects of the pancreatic beta cells. Several open questions remain in CHI research, such as the optimal treatment for the most common type of CHI, caused by mutations in the genes encoding ATP-sensitive potassium channels, and the molecular mechanisms of newly identified CHI genes. Answering these questions requires robust preclinical models, particularly since primary patient material is extremely scarce and accurate animal models are not available. In this short review, we explain why pluripotent stem cell derived islets present an attractive solution to these issues and outline the current progress in stem-cell based modeling of CHI. Stem cell derived islets enable the study of molecular mechanisms of CHI and the discovery of novel antihypoglycemic drugs, while also providing a valuable model to study the biology of variable functional states of beta cells.

## Introduction

Congenital hyperinsulinism (CHI), characterized by inappropriate insulin secretion from the pancreatic beta cells, is the most common cause of persistent childhood hypoglycemia. At least 15 causative genes have been identified (1), with 30-55% of patients remaining without a genetic diagnosis (2–5). Over 50% of all CHI patients (2, 5) carry a recessive loss-of-function mutation in the K_ATP_-channel genes *ABCC8* or *KCNJ11* (K_ATP_HI), which leads to abnormal membrane depolarization and constitutive insulin secretion. Clinically, this leads to severe hypoglycemia for which there is no optimal treatment. This represents an ongoing clinical challenge as the hypoglycemia is life-threatening in the first days of life and despite best contemporary treatment, many continue to suffer from learning difficulties in the long term (6–10). A robust preclinical model would be required for studies aiming to discover improved treatment options for K_ATP_HI and to pinpoint molecular mechanisms of rare newly identified forms of CHI.

Pluripotent stem cells (PSCs) represent the epiblast cells of the early embryo, capable of differentiation to any cell type in the human body. Tissue differentiated from PSCs holds enormous promise in regenerative medicine to replace or repair a damaged or degenerated organ. PSCs can also serve as a powerful research tool by allowing limitless generation of difficult-to-procure tissue and would thus serve as an attractive solution for preclinical study of CHI. Pluripotent stem cell derived islets (SC-islets) could replace or complement rodent models and primary patient islet tissue, which both have important inherent weaknesses. Rodent islets are structurally and physiologically different from human islets (11–14) and these differences have manifested in K_ATP_-channel knockout mouse models, which have presented with a much milder phenotype than the K_ATP_HI patients (15–17). The availability of healthy primary islets is limited and faces issues of variable *in vitro* function (www.epicore.ualberta.ca/isletcore/). The availability of CHI patient islets presents an additional challenge due to the rarity of the disease. The limited tissue availability challenges any study that requires large amounts of tissue, such as screening for novel pharmacotherapeutics.

This review focuses on the use of PSC-derived pancreatic islets (SC-islets) for preclinical study of congenital hyperinsulinism. We outline the practical necessities in setting up SC-islet models and aim to identify relevant questions for CHI research where the SC-islets are particularly powerful.

## SC-Islet Based Disease Modeling: Key Technologies and Current Progress

### Genome Editing of Pluripotent Stem Cells

Pluripotent stem cells (PSCs) are derived from two main sources: preimplantation embryos (embryonic stem cells, ESCs) (18) and somatic cells that have been reprogrammed back to pluripotent state by overexpression of key genes (induced pluripotent stem cells, iPSCs) (19). iPSCs reprogrammed from a patient sample carry the disease-causing mutations of that individual and should thus phenocopy the disease, such as CHI, when differentiated. A similarly differentiated healthy iPSC line would serve as a non-isogenic control for this type of approach. Theoretically, this offers a disease model without the need for genome editing. In practice however, the differences in the donor genetic background exert a high degree of influence on the differentiation efficiency of stem cell lines, at least in islet differentiation, making it difficult to conclude whether the detected differences between patient and healthy cell lines are due to the disease gene or a differentiation-related artefact. Thus, it is often more practical to correct the disease-causing mutation with genome editing tools to yield an isogenic control cell line. Isogenic controls offer a clean look into the disease phenotype without differences in the genetic background. Generating isogenic controls with genome editing of the PSCs can be considered as the ideal approach for the effective modeling of a genetic disease such as CHI.

Another approach to create isogenic cell lines is to engineer the disease-causing mutation in a healthy hPSC line. This approach is often more straightforward than the patient cell line approach because the differentiation protocol used to derive the SC-islets can be optimized for one cell line and all the interesting mutations can be engineered to it. Furthermore, from the genome editing point of view, it is easier to generate a knock-out than a knock-in. In correcting a patient line, a knock-in is always required, but for generating a disease cell line a simple knock-out is often enough since many diseases are caused by loss-of-function mutations.

Multiple technologies are available for the genome editing itself, but recently CRISPR-based technologies have started to dominate, due to their relative ease and high efficiency. The basic CRISPR system consists of a guide RNA, able to target the editing to a specific locus in the genome; a protein exhibiting nuclease activity such as Cas9 or Cas12a creating a double stand break; and an RNA template containing the mutation-corrected sequence or a sequence knocking out the healthy gene which can be read during homology directed repair of the double strand break. This basic system has been expanded and optimized further in many ways, as reviewed here (20). Regardless of the specifics, genome editing technology has matured to a state where generating disease relevant stem cell lines for further differentiation is practical.

### Differentiation of PSCs to Islets

Due to the curative potential of PSCs in cell replacement therapy for insulin deficient diabetes, enormous effort has been expended in developing protocols that can drive PSCs to differentiate *in vitro* to pancreatic islets (SC-islets). Starting with the breakthrough protocol published in 2006, showing for the first time that insulin positive cells can be differentiated from hPSCs through steps mimicking normal development (21), the progress in the field has been rapid. First evidence of glucose-regulated insulin release was provided in 2014 (22, 23), Since then, many further improvements have been made (24–26) and in the past two years the first protocols giving robust, dynamic glucose stimulated insulin secretion have been reported, achieving beta cell maturity at least in terms of insulin secretory function (27–35). This recent progress in the field has identified multiple conditions related to optimal late-stage maturation. These include keeping SC-islets appropriately sized by resizing or by culture format, keeping the proliferation rate low, normoglycemic culture conditions, lack of ALK5-inhibition, addition of WNT4, circadian entrainment, and reducing the number of unwanted cells that might compromise function on the islet level by sorting or by addition of aurora kinase inhibitor.

Given the recapitulation of the adult function in the state-of-the-art protocols and the fact that the protocols used to derive the mature SC-islet use the same signaling cues as the fetal islets during their *in vivo* development, SC-islets represent an excellent avenue for modeling CHI pathophysiology, including both developmental and insulin secretory defects. We have shown that all the main components of the stimulus-secretion coupling machinery of beta cells: metabolic processing of glucose, currents of the critical ion-channels, the insulin-secretion modulating amplifying mechanisms and the exocytosis machinery, are present in SC-islets (30). As most forms of CHI manifest in the neonatal period, achieving adult-like function might not even be necessary for some study questions. This is exemplified by existing SC-islet models for CHI, which have been successful even using less efficient differentiation protocols, as described in the following section.

### Stem Cell-Based Models for CHI

Thus far, two studies have taken advantage of the SC-islet differentiation and genome editing technologies in modeling K_ATP_-channel related CHI (K_ATP_HI) (36, 37), as summarized in **Table 1**. Guo and colleagues (36), used healthy hESCs and introduced a knockout of the *ABCC8* gene, encoding the K_ATP_-channel subunit SUR1. The *ABCC8* KO beta cells secreted around 2-fold more insulin *in vitro* and failed to respond to K_ATP_-channel acting pharmaceuticals. Their beta-like cells could be inhibited with octreotide, and to a lesser degree with nicorandil and nifedipine. Thus, they replicated the K_ATP_HI insulin secretion phenotype *in vitro*.

**Table 1:** Summary of published studies on stem cell based modeling of congenital hyperinsulinism.

***In vitro* phenotype** Studies 1&2	K_ATP_-mutant vs. control
Insulin secretion in low glucose	2-3 fold higher secretion
Response to diazoxide	No response vs. 50% reduction
***In vivo* phenotype** Study 2	
Fasting C-peptide	7 fold higher level in circulation
Fasting glucose	40% lower
**Developmental phenotype** Study 2	
Beta cell proportion	32% more beta cells
Beta cell proliferation	61% more proliferating beta cells

Study 1: Guo et al. Scientific Reports (36).

Study 2: Lithovius et al. Diabetologia (37).

We used iPSCs derived from a patient carrying the homozygous V187D-mutation (38) in the *ABCC8* gene and compared them to mutation-corrected controls (37). The *ABCC8* mutant beta-like cells secreted around 3-fold more insulin in low glucose compared to the corrected counterparts. They also failed to respond to K_ATP_-channel acting pharmaceuticals but could be inhibited with clonidine and EGTA. Upon transplantation and *in vivo* maturation under the kidney capsule of immunocompromised mice, the mutant grafts secreted 7-fold higher levels of human C-peptide and caused 38% lower blood glucose upon fasting than the control grafts. We could thus replicate the cardinal phenotypic features of K_ATP_HI both *in vitro* and *in vivo*. In addition to these features of the secretory function, we found that the K_ATP_-inactivation directed the development of endocrine cells towards beta cells at the expense of alpha cells in the *in vitro* differentiation. This may have been due to the increased proliferation we detected in the *ABCC8* mutant beta cells. The role of K_ATP_-channel inactivation in proliferation was also found in a previous study using pancreas-derived mesenchymal stem cells derived from a K_ATP_HI patient (39).

## Potential Research Avenues for SC-Islet CHI Models

### Development of Pharmacotherapy for Diazoxide-Resistant CHI

The first-line antihypoglycemic drug used in CHI, diazoxide, is an opener of the K_ATP_-channel and as such, most of the recessive K_ATP_HI patients are unresponsive to it (40, 41). Second-line treatments, such as somatostatin receptor agonists are widely used, but still the most severe patients must undergo pancreatectomy to control hypoglycemia. This is a suboptimal treatment which rarely results in euglycemia (42–44). There is an obvious need for more effective antihypoglycemic medication for diazoxide-resistant CHI.

SC-islets can be generated in limitless quantities and when derived with state-of-the-art differentiation protocols (27, 29, 30), they harbor the stimulus-secretion coupling machinery of adult primary islets: the K_ATP_-channel related triggering pathway, as well as the neurohormonal and metabolic amplifying pathways (30). This covers the key pathways that modulate insulin secretion and could thus serve as pharmacological targets.

**Figure 1:**
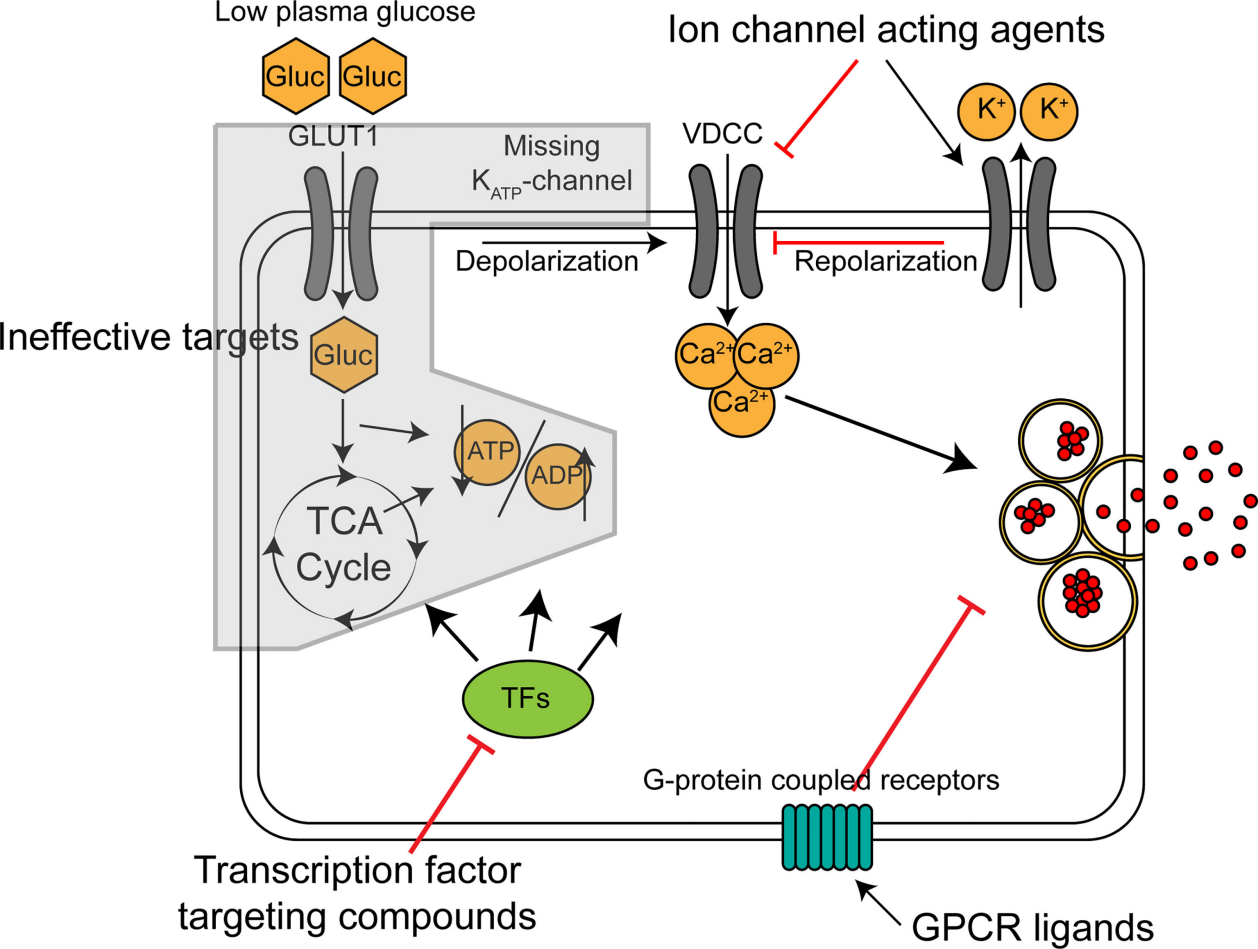
Mechanism of action for classes of potential antihypoglycemic agents for treatment of K_ATP_-channel related CHI. Broad, schematic depiction of insulin secretion machinery in K_ATP_-channel deficient beta cells in low glucose. GLUT1, glucose transporter; Gluc, glucose; VDCC, voltage-dependent calcium channel; TCA cycle, tricarboxylic acid cycle; TFs, transcription factors.

Potential antihypoglycemic targets on the beta cell include ion-channels, G-protein coupled receptors and transcription factors controlling beta cell function, as summarized in **Figure 1**. Depending on the genetic cause of CHI, some of these might be more advantageous than others. In the most common type of diazoxide resistant CHI, K_ATP_HI, insulin hypersecretion occurs because the K_ATP_-channel is inactive leading to constitutive depolarization and constantly elevated intracellular calcium (45). In this intracellular environment, a molecule acting upstream of the K_ATP_-channel in the stimulus-secretion coupling machinery (ie. glucose uptake, glycolysis and the TCA cycle) is likely to be ineffective. Potential antihypoglycemic ion-channel acting drugs would act by decreasing the membrane potential independently of the K_ATP_-channel (ie. by acting on K^+^, Na^+^ and Cl^-^ channels) or by reducing intracellular Ca^2+^, the final trigger of exocytosis. Nifedipine and other Ca^2+^ channel blockers have been used in the treatment of K_ATP_HI but have proven ineffective (46). Sikimic and colleagues identified DCEBIO, acting as an agonist of the repolarizing K_Ca3.1_ channel, as a molecule abolishing the glucose induced Ca^2+^ oscillations in two K_ATP_HI islet preparations (47). A potential downside with ion-channel acting molecules is that many of the target channels are also expressed in non-islet cells, such as cardiomyocytes and neurons, increasing the likelihood of serious side effects.

Several islet specific G-protein coupled receptors (GPCRs) have been identified (48). The role of these receptors in beta cells is to fine-tune the glucose-stimulated insulin secretion by affecting the amplifying pathways. They exert their action through cAMP and other second messengers, which sensitize or desensitize insulin granule exocytosis. Human islets express around 300 GPCRs (49). Highly expressed ones include receptors for the other islet hormones, glucagon and somatostatin, and the gut-derived incretins, GLP1 and GIP, but many beta cell GPCRs also have no known ligand (orphan receptors). This offers the potential for novel antihypoglycemic compounds to be found by screening. Many of the most potent GPCR-coupled amplifying pathways, such as the GLP1-receptor coupled pathway, are primarily stimulated only when the beta cells are already triggered by the K_ATP_-channel closure and subsequent influx of calcium, restricting their activity mostly to high glucose conditions (50). The K_ATP_HI cells are constantly in the state of high [Ca^2+^]_i_, which should allow the inappropriate activation of these pathways even in low glucose. Targeting GPCRs in K_ATP_HI has been shown to be effective by the well-established use of somatostatin receptor agonists such as octreotide. More recently, studies by De León and associates have identified the GLP1-receptor inverse agonist exendin-(9-39) as an effective antihypoglycemic agent in K_ATP_-KO mice (51) and in adult K_ATP_HI patients (52).

Transcription factors controlling beta cell insulin secretion could serve as a third group of targets for antihypoglycemic medication. Senniappan and colleagues demonstrated the validity of this approach using mTOR inhibitor rapamycin on diazoxide-resistant CHI patients (53). Since the initial report however, the use of rapamycin has been questioned due to low efficacy and high incidence of serious side effects (54, 55). These issues are likely due to mTOR being an important regulator of wide variety of cellular processes in most tissues, and as such, an agent acting on a transcription factor more specifically controlling beta cell insulin secretion related gene expression would be more ideal. Despite the existence of several beta cell-specific transcription factors that control the expression of insulin secretory machinery genes (e.g., MAFA or RFX6), small molecules specifically targeting them are missing, and thus the ion-channel and GPCR-related strategies are likely to provide more accessible targets.

The most clinically relevant parameter for identifying the potential drug would be its effectiveness in reducing the CHI SC-islets’ insulin secretion in low glucose. Measuring just intracellular calcium fluxes with a dye or a genetically encoded sensor could lead to failure in identifying a potential drug if the drug acts on the amplifying mechanisms of insulin secretion, whose activity does not result in further calcium fluxes. Any candidate identified in the *in vitro* screens should be validated *in vivo*. The first line strategy for this is to use CHI SC-islet grafts, which lead to hypoglycemia in the recipient mice (37). Conceivably measurements of mouse blood glucose and C-peptide secreted by the graft in drug-treated and non-treated mice engrafted with CHI SC-islets should reveal the effectiveness of a candidate molecule *in vivo*.

### Discovery of Novel CHI Pathomechanisms

Around 50% of new CHI patients do not present a mutation in the genes previously identified as causative for CHI (2). Unraveling of the exact pathogenic mechanism should direct selection of the most appropriate treatment for each patient. Pinpointing the molecular mechanism could also aid discovery of antihypoglycemic pharmaceuticals and shed light to the role of the identified genes in the regulation of beta cell insulin secretion. Answering these questions requires a model system with sufficient fidelity to capture disease pathophysiology on many levels. SC-islets have been used to discover the molecular mechanism of multiple genes causing different types of monogenic diabetes, ranging from mechanisms related to beta cell development (56–58) to function (59, 60) and degeneration (61–63), as reviewed recently (64). In the case of novel CHI genes, several parameters can be used to unravel disease mechanisms. These include, at least, development of the endocrine populations during differentiation; insulin secretion under different stimuli *in vitro*; transcriptomic, epigenomic proteomic, and dynamic metabolomic studies. The stem cell based approach can provide even the large amounts of tissue required for these analyses.

### The Use of CHI SC-Islets as a Model for Chronic Beta-Cell Hyperfunctionality and Glucotoxicity

The beta cell is highly specialized, focusing on the production and secretion of insulin. It is understandable that CHI mutations, which by definition accelerate this process, have profound consequences for the cellular biology of the beta cells. Huopio and colleagues established that, in addition to causing hypoglycemia in infancy (65), dominant K_ATP_HI mutations predispose to T2DM later in life (66), providing clinical evidence for the detrimental consequences of long-term beta cell hyperactivity. Similar T2DM predisposing effect has been discovered in carriers of activating glucokinase mutations (GCK-HI) (67, 68).

Li and colleagues found that diverse cellular functions are disturbed or altered in CHI patient beta cells lacking K_ATP_-channels, including glucose dependent metabolic pathways, expression of key transcription factors and receptors and the regulation of cell cycle (69). Additionally, the chronically elevated [Ca^2+^]_i_ characteristic of K_ATP_HI and GCK-HI compromises beta cell identity in (70) and causes double strand breaks and p53 activation (71). Related to these findings, the increased workload of CHI beta cells initially increases their proliferation and mass while later leading to beta cell dysfunction and apoptosis *via* glucotoxicity (71–74), thus paralleling the natural course of beta cells in type 2 diabetes. These examples highlight the possibility of using CHI SC-islets as a model to discover further consequences of the influence of chronically altered beta cell functional states on their biology. Indeed, we could capture the initially increased beta cell mass and proliferation *in vitro*, while the proliferation normalized *in vivo* (37). Again, the possibility for isogenic comparison between healthy and CHI SC-islets provides a specific means to link any alterations to specific mutations.

## Conclusions

Stem cell derived islets represent a powerful tool for modeling diseases of the pancreatic beta cell, due to the potential to produce them in limitless quantities with high consistency and with high disease phenotype fidelity. In the case of CHI, the SC-islets can be harnessed to discover novel antihypoglycemic medications, to study molecular mechanisms of newly discovered CHI genes and to study the basic biology of a hyperactive beta cell. Thus, we believe that modeling of CHI with SC-islets will serve as a critical next step required for the development of specific and efficient antihypoglycemic drugs.

## Author Contributions

VL wrote the initial draft and edited the manuscript. TO edited the manuscript and provided resources. All authors contributed to the article and approved the submitted version.

## Funding

The studies in the Otonkoski laboratory were supported by the Academy of Finland (Center of Excellence MetaStem, grant no. 312437), the Novo Nordisk Foundation and the Sigrid Jusélius Foundation.

## Conflict of Interest

The authors declare that the research was conducted in the absence of any commercial or financial relationships that could be construed as a potential conflict of interest.

## Publisher’s Note

All claims expressed in this article are solely those of the authors and do not necessarily represent those of their affiliated organizations, or those of the publisher, the editors and the reviewers. Any product that may be evaluated in this article, or claim that may be made by its manufacturer, is not guaranteed or endorsed by the publisher.

## References

[1] Gϋemes M Rahman SA Kapoor RR Flanagan S Houghton JAL Misra S. Hyperinsulinemic Hypoglycemia in Children and Adolescents: Recent Advances in Understanding of Pathophysiology and Management. Rev Endocr Metab Disord (2020) 21(4):577–97. doi: 10.1007/s11154-020-09548-7 PMC7560934 32185602

[2] Kapoor RR Flanagan SE Arya VB Shield JP Ellard S Hussain K. Clinical and Molecular Characterisation of 300 Patients With Congenital Hyperinsulinism. Eur J Endocrinol (2013) 168(4):557–645. doi: 10.1530/EJE-12-0673 23345197 PMC3599069

[3] Männistö JME Maria M Raivo J Kuulasmaa T Otonkoski T Huopio H. Clinical and Genetic Characterization of 153 Patients With Persistent or Transient Congenital Hyperinsulinism. J Clin Endocrinol Metab (2020) 105(4):E1686–945. doi: 10.1210/clinem/dgz271 32170320

[4] Rosenfeld E Ganguly A de Leon DD. Congenital Hyperinsulinism Disorders: Genetic and Clinical Characteristics. Am J Med Genet Part C: Semin Med Genet (2019) 181(4):682–925. doi: 10.1002/ajmg.c.31737 31414570 PMC7229866

[5] Snider KE Becker S Boyajian L Shyng SL MacMullen C Hughes N. Genotype and Phenotype Correlations in 417 Children With Congenital Hyperinsulinism. J Clin Endocrinol Metab (2013) 98(2):355–63. doi: 10.1210/jc.2012-2169 PMC3565119 23275527

[6] Muukkonen L Männistö J Jääskeläinen J Hannonen R Huopio H. The Effect of Hypoglycaemia on Neurocognitive Outcome in Children and Adolescents With Transient or Persistent Congenital Hyperinsulinism. Dev Med Child Neurol (2019) 61(4):451–575. doi: 10.1111/dmcn.14039 30246438

[7] Roeper M Dafsari RS Hoermann H Mayatepek E Kummer S Meissner T. Risk Factors for Adverse Neurodevelopment in Transient or Persistent Congenital Hyperinsulinism. Front Endocrinol (2020) 11:580642. doi: 10.3389/fendo.2020.580642 PMC7793856 33424766

[8] Shah R Harding J Brown J Mckinlay C. Neonatal Glycaemia and Neurodevelopmental Outcomes: A Systematic Review and Meta-Analysis. Neonatology (2019) 115(2):116–265. doi: 10.1159/000492859 30408811

[9] Lord K Radcliffe J Gallagher PR Adzick NS Stanley CA de León DD. High Risk of Diabetes and Neurobehavioral Deficits in Individuals With Surgically Treated Hyperinsulinism. J Clin Endocrinol Metab (2015) 100(11):4133–395. doi: 10.1210/jc.2015-2539 PMC4702456 26327482

[10] Helleskov A Melikyan M Globa E Shcherderkina I Poertner F Larsen AM. Both Low Blood Glucose and Insufficient Treatment Confer Risk of Neurodevelopmental Impairment in Congenital Hyperinsulinism: A Multinational Cohort Study. Front Endocrinol (2017) 8:156. doi: 10.3389/fendo.2017.00156 PMC5502348 28740482

[11] Cabrera O Berman DM Kenyon NS Ricordi C Berggren P-O Caicedo A. The Unique Cytoarchitecture of Human Pancreatic Islets Has Implications for Islet Cell Function. Proc Natl Acad Sci USA (2006) 103(7):2334–95. doi: 10.1073/pnas.0510790103 PMC1413730 16461897

[12] Nair G Hebrok M. Islet Formation in Mice and Men: Lessons for the Generation of Functional Insulin-Producing β-Cells From Human Pluripotent Stem Cells. Curr Opin Genet Dev (2015) 32:171–80. doi: 10.1016/j.gde.2015.03.004 PMC4523641 25909383

[13] Balboa D Otonkoski T. Human Pluripotent Stem Cell Based Islet Models for Diabetes Research. Best Pract Res: Clin Endocrinol Metab (2015) 29(6):899–9095. doi: 10.1016/j.beem.2015.10.012 26696518

[14] Rodriguez-Diaz R Molano RD Weitz JR Abdulreda MH Berman DM Leibiger B. Paracrine Interactions Within the Pancreatic Islet Determine the Glycemic Set Point. Cell Metab (2018) 27(3):549–58.e4. doi: 10.1016/j.cmet.2018.01.015 29514065 PMC5872154

[15] Seghers V Nakazaki M DeMayo F Aguilar-Bryan L Bryan J. Sur1 Knockout Mice. A Model for K(ATP) Channel-Independent Regulation of Insulin Secretion. J Biol Chem (2000) 275(13):9270–7. doi: 10.1074/jbc.275.13.9270 10734066

[16] Miki T Nagashima K Tashiro F Kotake K Yoshitomi H Tamamoto A. Defective Insulin Secretion and Enhanced Insulin Action in K ATP Channel-Deficient Mice. Biochemistry (1998) 95:10402–6. doi: 10.1073/pnas.95.18.10402 PMC27906 9724715

[17] Shiota C Larsson O Shelton K Shiota M Efanov A Hoy M. Sulfonylurea Receptor Type 1 Knock-Out Mice Have Intact Feeding-Stimulated Insulin Secretion Despite Marked Impairment in Their Response to Glucose. J Biol Chem (2002) 277:37176–83. doi: 10.1074/jbc.M206757200 12149271

[18] Thomson JA. Embryonic Stem Cell Lines Derived From Human Blastocysts. Science (1998) 282(5391):1145–7. doi: 10.1126/science.282.5391.1145 9804556

[19] Takahashi K Tanabe K Ohnuki M Narita M Ichisaka T Tomoda K. Induction of Pluripotent Stem Cells From Adult Human Fibroblasts by Defined Factors. Cell (2007) 131(5):861–72. doi: 10.1016/j.cell.2007.11.019 18035408

[20] Anzalone AV Koblan LW Liu DR. Genome Editing With CRISPR–Cas Nucleases, Base Editors, Transposases and Prime Editors. Nat Biotechnol (2020) 38(7):824–44. doi: 10.1038/s41587-020-0561-9 32572269

[21] D’Amour KA Bang AG Eliazer S Kelly OG Agulnick AD Smart NG. Production of Pancreatic Hormone-Expressing Endocrine Cells From Human Embryonic Stem Cells. Nat Biotechnol (2006) 24(11):1392–401. doi: 10.1038/nbt1259 17053790

[22] Rezania A Bruin JE Arora P Rubin A Batushansky I Asadi A. Reversal of Diabetes With Insulin-Producing Cells Derived in Vitro From Human Pluripotent Stem Cells. Nat Biotechnol (2014) 32(11):1121–33. doi: 10.1038/nbt.3033 25211370

[23] Pagliuca FW Millman JR Gürtler M Segel M Van Dervort A Ryu JH. Generation of Functional Human Pancreatic β Cells. In Vitro Cell (2014) 159(2):428–39. doi: 10.1016/j.cell.2014.09.040 PMC4617632 25303535

[24] Nostro MC Sarangi F Yang C Holland A Elefanty AG Stanley EG. Efficient Generation of NKX6-1+ Pancreatic Progenitors From Multiple Human Pluripotent Stem Cell Lines. Stem Cell Rep (2015) 4(4):591–6045. doi: 10.1016/j.stemcr.2015.02.017 PMC4400642 25843049

[25] Russ HA Parent AV Ringler JJ Hennings TG Nair GG Shveygert M. Controlled Induction of Human Pancreatic Progenitors Produces Functional Beta-Like Cells *In Vitro* . EMBO J (2015) 34(13):e201591058. doi: 10.15252/embj.201591058 PMC4516429 25908839

[26] Toyoda T Kimura A Tanaka H Ameku T Mima A Hirose Y. Rho-Associated Kinases and Non-Muscle Myosin IIs Inhibit the Differentiation of Human IPSCs to Pancreatic Endoderm. Stem Cell Rep (2017) 9(2):419–28. doi: 10.1016/j.stemcr.2017.07.005 PMC5550204 28793244

[27] Velazco-Cruz L Song J Maxwell KG Goedegebuure MM Augsornworawat P Hogrebe NJ. Acquisition of Dynamic Function in Human Stem Cell-Derived Beta Cells. Stem Cell Rep (2019) 12(2):351–65. doi: 10.1016/j.stemcr.2018.12.012 PMC6372986 30661993

[28] Hogrebe NJ Augsornworawat P Maxwell KG Velazco-Cruz L Millman JR. Targeting the Cytoskeleton to Direct Pancreatic Differentiation of Human Pluripotent Stem Cells. Nat Biotechnol (2020) 38(4):460–70. doi: 10.1038/s41587-020-0430-6 PMC7274216 32094658

[29] Nair GG Liu JS Russ HA Tran S Saxton MS Chen R. Recapitulating Endocrine Cell Clustering in Culture Promotes Maturation of Human Stem-Cell-Derived β Cells. Nat Cell Biol (2019) 21(2):263–74. doi: 10.1038/s41556-018-0271-4 PMC6746427 30710150

[30] Balboa D Barsby T Lithovius V Saarimäki-vire J Omar-hmeadi M Dyachok O. Functional, Metabolic and Transcriptional Maturation of Human Pancreatic Islets Derived From Stem Cell. BioRxiv (2021) 447439:1–20. doi: 10.1038/s41587-022-01219-z PMC9287162 35241836

[31] Yoshihara E Connor CO Gasser E Wei Z Oh TG Tseng TW. Immune-Evasive Human Islet-Like Organoids Ameliorate Diabetes. Nature (2020) 586:606–11. doi: 10.1038/s41586-020-2631-z PMC7872080 32814902

[32] Helman A Cangelosi AL Davis JC Straubhaar JR Sabatini DM Melton DA. Article A Nutrient-Sensing Transition at Birth Triggers Glucose-Responsive Insulin Secretion Ll Article A Nutrient-Sensing Transition at Birth Triggers Glucose-Responsive Insulin Secretion. Cell Metab (2020) 31(5):1004–16.e5. doi: 10.1016/j.cmet.2020.04.004 32375022 PMC7480404

[33] Davis JC Alves TC Helman A Liu DR Kibbey RG Melton DA. Article Glucose Response by Stem Cell-Derived B Cells In Vitro Is Inhibited by a Bottleneck in Glycolysis Ll Ll Glucose Response by Stem Cell-Derived B Cells *In Vitro* Is Inhibited by a Bottleneck in Glycolysis. CellReports (2020) 31(6):107623. doi: 10.1016/j.celrep.2020.107623 PMC7433758 32402282

[34] Alvarez-Dominguez JR Donaghey J Rasouli N Kenty JHR Helman A Charlton J. Circadian Entrainment Triggers Maturation of Human. In Vitro Islets Cell Stem Cell (2020) 26(1):108–22.e10. doi: 10.1016/j.stem.2019.11.011 31839570

[35] Mahaddalkar PU Scheibner K Pfluger S Ansarullah MS Beckenbauer J Irmler M. Generation of Pancreatic β Cells From CD177+ Anterior Definitive Endoderm. Nat Biotechnol (2020) 38(9):1061–72. doi: 10.1038/s41587-020-0492-5 32341565

[36] Guo D Liu H Ru A Gao GE Nasir A. Modeling Congenital Hyperinsulinism With ABCC8 - Deficient Human Embryonic Stem Cells Generated by CRISPR / Cas9. Sci Rep (2017) 7(3156):1–8. doi: 10.1038/s41598-017-03349-w 28600547 PMC5466656

[37] Lithovius V Saarimäki-Vire J Balboa D Ibrahim H Montaser H Barsby T. SUR1-Mutant iPS Cell-Derived Islets Recapitulate the Pathophysiology of Congenital Hyperinsulinism. Diabetologia (2021) 64(3):630–40. doi: 10.1007/s00125-020-05346-7 33404684

[38] Otonkoski T Ämmälä C Huopio H Cote GJ Chapman J Cosgrove K. A Point Mutation Inactivating the Sulfonylurea Receptor Causes the Severe Form of Persistent Hyperinsulinemic Hypoglycemia of Infancy in Finland. Diabetes (1999) 48:408–15. doi: 10.2337/diabetes.48.2.408 10334322

[39] Kellaway SG Mosinska K Mohamed Z Ryan A Richardson S Newbould M. Increased Proliferation and Altered Cell Cycle Regulation in Pancreatic Stem Cells Derived From Patients With Congenital Hyperinsulinism. (2019) PLoS ONE 14(9):e0222350. doi: 10.1371/journal.pone.0222350 PMC6746350 31525223

[40] Banerjee I Salomon-Estebanez M Shah P Nicholson J Cosgrove KE Dunne MJ. Therapies and Outcomes of Congenital Hyperinsulinism-Induced Hypoglycaemia. Diabetic Med (2019) 36(1):9–21. doi: 10.1111/dme.13823 30246418 PMC6585719

[41] Henquin JC Nenquin M Sempoux C Guiot Y Bellanné-Chantelot C Otonkoski T. In Vitro Insulin Secretion by Pancreatic Tissue From Infants With Diazoxide-Resistant Congenital Hyperinsulinism Deviates From Model Predictions. J Clin Invest (2011) 121(10):3932–42. doi: 10.1172/JCI58400 PMC3195476 21968111

[42] Adzick NS de Leon DD States LJ Lord K Bhatti TR Becker SA. Surgical Treatment of Congenital Hyperinsulinism: Results From 500 Pancreatectomies in Neonates and Children. J Pediatr Surg (2019) 54(1):27–32. doi: 10.1016/j.jpedsurg.2018.10.030 30343978 PMC6339589

[43] Beltrand J Caquard M Arnoux J-B Laborde K Velho G Verkarre V. Glucose Metabolism in 105 Children and Adolescents After Pancreatectomy for Congenital Hyperinsulinism. Diabetes Care (2012) 35(2):198–203. doi: 10.2337/dc11-1296 22190679 PMC3263917

[44] Arya VB Senniappan S Demirbilek H Alam S Flanagan SE Ellard S. Pancreatic Endocrine and Exocrine Function in Children Following Near-Total Pancreatectomy for Diffuse Congenital Hyperinsulinism. PloS One (2014) 9(5):4–9. doi: 10.1371/journal.pone.0098054 PMC4026387 24840042

[45] Thomas PM Cote GJ Wohilk N Haddad B Mathew PM Rabl W. Mutations in the Sulfonylurea Receptor Gene in Familial Persistent Hyperinsulinemic Hypoglycemia of Infancy. Science (1995) 268:426–29. doi: 10.1126/science.7716548 7716548

[46] Güemes M Shah P Silvera S Morgan K Gilbert C Hinchey L. Assessment of Nifedipine Therapy in Hyperinsulinemic Hypoglycemia Due to Mutations in the Abcc8 Gene. J Clin Endocrinol Metab (2017) 102(3):822–30. doi: 10.1210/jc.2016-2916 27898257

[47] Sikimic J Hoffmeister T Gresch A Kaiser J Barthlen W Wolke C. Possible New Strategies for the Treatment of Congenital Hyperinsulinism. Front Endocrinol (2020) 11:545638. doi: 10.3389/fendo.2020.545638 PMC7653201 33193079

[48] Regard JB Sato IT Coughlin SR. Anatomical Profiling of G Protein-Coupled Receptor Expression. Cell (2008) 135(3):561–71. doi: 10.1016/j.cell.2008.08.040 PMC2590943 18984166

[49] Amisten S Salehi A Rorsman P Jones PM Persaud SJ. An Atlas and Functional Analysis of G-Protein Coupled Receptors in Human Islets of Langerhans. Pharmacol Ther (2013) 139(3):359–91. doi: 10.1016/j.pharmthera.2013.05.004 23694765

[50] Gromada J Bokvist K Ding WG Holst JJ Nielsen JH Rorsman P. Glucagon-Like Peptide 1(7-36) Amide Stimulates Exocytosis in Human Pancreatic β-Cells by Both Proximal and Distal Regulatory Steps in Stimulus- Secretion Coupling. Diabetes (1998) 47(1):57–65. doi: 10.2337/diab.47.1.57 9421375

[51] de León DD Li C Delson MI Matschinsky FM Stanley CA Stoffers DA. Exendin-(9-39) Corrects Fasting Hypoglycemia in SUR-1-/- Mice by Lowering Camp in Pancreatic β-Cells and Inhibiting Insulin Secretion. J Biol Chem (2008) 283(38):25786–935. doi: 10.1074/jbc.M804372200 PMC3258866 18635551

[52] Calabria AC Li C Gallagher PR Stanley CA de León DD. GLP-1 Receptor Antagonist Exendin-(9-39) Elevates Fasting Blood Glucose Levels in Congenital Hyperinsulinism Owing to Inactivating Mutations in the ATP-Sensitive K+ Channel. Diabetes (2012) 61(10):2585–91. doi: 10.2337/db12-0166 PMC3447900 22855730

[53] Senniappan S Alexandrescu S Tatevian N Shah P Arya V Flanagan S. Sirolimus Therapy in Infants With Severe Hyperinsulinemic Hypoglycemia. N Engl J Med (2014) 370(12):1131–37. doi: 10.1056/NEJMoa1310967 24645945

[54] Szymanowski M Estebanez MS Padidela R Han B Mosinska K Stevens A. MTOR Inhibitors for the Treatment of Severe Congenital Hyperinsulinism: Perspectives on Limited Therapeutic Success. J Clin Endocrinol Metab (2016) 101(12):4719–29. doi: 10.1210/jc.2016-2711 27691052

[55] Güemes M Dastamani A Ashworth M Morgan K Ellard S Flanagan SE. Sirolimus: Efficacy and Complications in Children With Hyperinsulinemic Hypoglycemia: A 5-Year Follow-Up Study. J Endocr Soc (2019) 3(4):699–713. doi: 10.1210/js.2018-00417 30882046 PMC6411415

[56] Saarimäki-Vire J Balboa D Russell MA Saarikettu J Kinnunen M Keskitalo S. An Activating STAT3 Mutation Causes Neonatal Diabetes Through Premature Induction of Pancreatic Differentiation. Cell Rep (2017) 19(2):281–94. doi: 10.1016/j.celrep.2017.03.055 28402852

[57] Teo AKK Lau HH Valdez IA Dirice E Tjora E Raeder H. Early Developmental Perturbations in a Human Stem Cell Model of MODY5/HNF1B Pancreatic Hypoplasia. Stem Cell Rep (2016) 6(3):357–67. doi: 10.1016/j.stemcr.2016.01.007 PMC4788763 26876668

[58] Vethe H Ghila L Berle M Hoareau L Haaland Ø Scholz H. The Effect of Wnt Pathway Modulators on Human IPSC-Derived Pancreatic Beta Cell Maturation. Front Endocrinol (2019) 10:293. doi: 10.3389/fendo.2019.00293 PMC6518024 31139151

[59] Balboa D Saarimäki-Vire J Borshagovski D Survila M Lindholm P Galli E. Insulin Mutations Impair Beta-Cell Development in a Patient-Derived iPSC Model of Neonatal Diabetes. ELife (2018) 7:e38519. doi: 10.7554/eLife.38519 30412052 PMC6294552

[60] Zeng H Guo M Zhou T Tan L Chong CN Zhang T. An Isogenic Human ESC Platform for Functional Evaluation of Genome-Wide-Association-Study-Identified Diabetes Genes and Drug Discovery. Cell Stem Cell (2016) 19(3):326–40. doi: 10.1016/j.stem.2016.07.002 PMC5924691 27524441

[61] de Franco E Lytrivi M Ibrahim H Montaser H Wakeling MN Fantuzzi F. YIPF5 Mutations Cause Neonatal Diabetes and Microcephaly Through Endoplasmic Reticulum Stress. J Clin Invest (2020) 130(12):6338–53. doi: 10.1172/JCI141455 PMC7685733 33164986

[62] Montaser H Patel KA Balboa D Ibrahim H Lithovius V Näätänen A. Loss of MANF Causes Childhood-Onset Syndromic Diabetes Due to Increased Endoplasmic Reticulum Stress. Diabetes (2021) 70(4):1006–18. doi: 10.2337/db20-1174 PMC7610619 33500254

[63] Maxwell KG Augsornworawat P Velazco-Cruz L Kim MH Asada R Hogrebe NJ. Gene-Edited Human Stem Cell–Derived β Cells From a Patient With Monogenic Diabetes Reverse Preexisting Diabetes in Mice. Sci Trans Med (2020) 12(540):eaax9106. doi: 10.1126/scitranslmed.aax9106 PMC7233417 32321868

[64] Balboa D Iworima DG Kieffer TJ. Human Pluripotent Stem Cells to Model Islet Defects in Diabetes. Front Endocrinol (Lausanne) (2021) 12:1–19. doi: 10.3389/fendo.2021.642152 PMC8020750 33828531

[65] Huopio H Reimann F Ashfield R Komulainen J Lenko H-l Rahier J. Dominantly Inherited Hyperinsulinism Caused by a Mutation in the Sulfonylurea Receptor Type 1. J Clin Invest (2000) 106: (7):897–906. doi: 10.1172/JCI9804 11018078 PMC381424

[66] Huopio H Otonkoski T Vauhkonen I Reimann F Ashcroft FM Laakso M. A New Subtype of Autosomal Dominant Diabetes Attributable to a Mutation in the Gene for Sulfonylurea Receptor 1. Lancet (2003) 361(9354):301–7. doi: 10.1016/S0140-6736(03)12325-2 12559865

[67] Langer S Waterstradt R Hillebrand G Santer R Baltrusch S. The Novel GCK Variant P.Val455Leu Associated With Hyperinsulinism Is Susceptible to Allosteric Activation and Is Conducive to Weight Gain and the Development of Diabetes. Diabetologia (2021) 64(12):2687–700. doi: 10.1007/s00125-021-05553-w PMC8563668 34532767

[68] Glaser B Kesavan P Heyman M Davis E Cuesta A Buchs A. Familial Hyperinsulinism Caused by an Activating Glucokinase Mutation. N Engl J Med (1998) 338(4):226–30. doi: 10.1056/NEJM199801223380404 9435328

[69] Li C Ackermann AM Boodhansingh KE Bhatti TR Liu C Schug J. Functional and Metabolomic Consequences of ATP-Dependent Potassium Channel Inactivation in Human Islets. Diabetes (2017) 66:db170029. doi: 10.2337/db17-0029 PMC5482088 28442472

[70] Stancill JS Cartailler JP Clayton HW O’Connor JT Dickerson MT Dadi PK. Chronic β-Cell Depolarization Impairs β-Cell Identity by Disrupting a Network of Ca2+-Regulated Genes. Diabetes (2017) 66(8):2175–87. doi: 10.2337/db16-1355 PMC5521870 28550109

[71] Tornovsky-Babeay S Dadon D Ziv O Tzipilevich E Kadosh T Haroush RS-B. Type 2 Diabetes and Congenital Hyperinsulinism Cause DNA Double-Strand Breaks and P53 Activity in β Cells. Cell Metab (2014) 19(1):109–21. doi: 10.1016/j.cmet.2013.11.007 24332968

[72] Tornovsky-Babeay S Weinberg-Corem N Schyr RB-H Avrahami D Lavi J Feleke E. Biphasic Dynamics of Beta Cell Mass in a Mouse Model of Congenital Hyperinsulinism: Implications for Type 2 Diabetes. Diabetologia (2021) 64(5):1133–43. doi: 10.1007/s00125-021-05390-x PMC8117185 33558985

[73] Kassem S Bhandari S Rodríguez-Bada P Motaghedi R Heyman M García-Gimeno MA. Large Islets, Beta-Cell Proliferation, and a Glucokinase Mutation. N Engl J Med (2010) 362(14):1348–50. doi: 10.1056/nejmc0909845 20375417

[74] Kassem SA Ariel I Thornton PS Scheimberg I Glaser B. Beta-Cell Proliferation and Apoptosis in the Developing Normal Human Pancreas and in Hyperinsulinism of Infancy. Diabetes (2000) 49(8):1325–33. doi: 10.2337/diabetes.49.8.1325 10923633

